# Research on Modification Technology of Laser Cladding Stellite6/Cu Composite Coating on the Surface of 316L Stainless Steel Plow Teeth

**DOI:** 10.3390/mi16070827

**Published:** 2025-07-20

**Authors:** Wenhua Wang, Qilang He, Wenqing Shi, Weina Wu

**Affiliations:** 1 School of Electronic and Information Engineering, Guangdong Ocean University, Zhanjiang 524088, China; wangwh@gdou.edu.cn (W.W.); gdouheql@163.com (Q.H.); 2 School of Materials Science and Engineering, Guangdong Ocean University, Yangjiang 529500, China; afjswq@163.com; 3 School of Mathematics and Computer Science, Guangdong Ocean University, Zhanjiang 524088, China

**Keywords:** laser cladding technology, surface modification, 316L plow teeth of plow loosening machine, Stellite6/Cu composite coatings

## Abstract

Plow loosening machines are essential agricultural machinery in the agricultural production process. Improving the surface strengthening process and extending the working life of the plow teeth of the plow loosening machine are of great significance. In this paper, the preparation of Stellite6/Cu composite coating on the surface of 316L steel substrate intended for strengthening the plow teeth of a plow loosening machine using laser cladding technology was studied. The influence of different laser process parameters on the microstructure and properties of Stellite6/Cu composite coating was investigated. The composite coating powder was composed of Stellite6 powder with a different weight percent of copper. Microstructural analysis, phase composition, elemental distribution, microhardness, wear resistance, and corrosion resistance of the composite coatings on the plow teeth were analyzed using scanning electron microscopy (SEM), X-ray diffraction (XRD), microhardness testing, energy dispersive spectroscopy (EDS), friction and wear testing, and electrochemical workstation measurements. The results showed that (1) When the laser power was 1000 W, the average hardness of the prepared Stellite6/Cu composite layer achieved the highest hardness, approximately 1.36 times higher than the average hardness of the substrate, and the composite coating prepared exhibited the best wear resistance; (2) When the scanning speed was 800 mm/min, the composite coating exhibited the lowest average friction coefficient and the optimal corrosion resistance in a 3.5% wt.% NaCl solution with a self-corrosion current density of −7.55 µA/cm^2^; (3) When the copper content was 1 wt.%, the composite coating achieved the highest average hardness with 515.2 HV, the lowest average friction coefficient with 0.424, and the best corrosion resistance with a current density of −8.878 µA/cm^2^.

## 1. Introduction

Laser cladding is an advanced and efficient technology for surface strengthening and remanufacturing of metal parts. This technique enables the formation of high-performance coatings through precise control of process parameters such as laser power and scanning speed, significantly enhancing substrate surface quality and performance, thereby extending component service life [1,2,3]. 316L stainless steel is widely used in the manufacturing of mechanical equipment components in many industries within agriculture, such as the plow teeth of plow loosening machines, due to its excellent corrosion resistance and good mechanical properties [4]. The plow loosening machine employs the traction force of the tractor to deeply loosen soil with the plow teeth; the process is mainly used in agricultural applications to strip and loosen frozen soil, soft mineral rocks, and medium-hard fractured mineral rocks. Deep loosening technology has been shown to significantly increase crop yield, particularly for deep-rooted crops, representing an important yield-enhancing technology [5]. In modern agricultural production, agricultural compound fertilizer is a necessity for many crops. Agricultural compound fertilizers and some soils contain halogen elements, and there is often a certain amount of water retained in the soil. Working in the soil for a long time can easily cause corrosion of the plow teeth [6,7,8]. At the same time, due to the frequent external impact on the plow teeth during operation, serious wear and tear can occur, resulting in reduced production efficiency and increased cultivation costs [5,7,8]. In order to enhance the wear and corrosion resistance and the other performance of the parts before service, the laser cladding modification technology is usually employed to melt high-temperature alloy powder onto the surface of 316L stainless steel substrate.

Copper is a material with high thermal conductivity, high electrical conductivity, good ductility, and corrosion resistance. As a cost-effective and widely applicable metal material, it is suitable for electrical and pipeline engineering. However, it often appears in the form of copper/steel composite structures in practical applications due to its low strength [9]. In response to the deficiencies in copper alloy applications, various methods enhancing the performance have been developed. Among these, electroplating and thermal spraying processes are less favored due to their susceptibility to peeling of the coating [10,11]. Laser cladding technology has become the research focus because it can not only maintain the excellent properties of the substrate material itself but also improve the surface properties of the material and reduce production costs by preparing wear-resistant and corrosion-resistant coatings on the surface of the substrate material [1,2,3,12,13]. Guo et al. [14] prepared Inconel 625 coating on the surface of 30CrMo steel plates by laser cladding, focusing on the effects of scanning speed and laser power and the effective height of the cladding layer. The coating quality was analyzed using an X-ray diffractometer, optical microscope, etc. The high laser reflectivity and thermal conductivity of copper can lead to poor forming quality and limited performance improvement of substrates during laser cladding. Therefore, how to improve the forming effect and maintain the excellent performance of the cladding layer is still a widespread concern in practical applications [15,16].

Cobalt-based alloys exhibit superior corrosion resistance [17,18,19] and high temperature resistance [20] compared to nickel-based and iron-based alloys, often making cobalt-based coatings prepared using laser cladding technology perform outstandingly in harsh application environments [21,22,23]. Due to its excellent corrosion resistance in chloride ion environments, 316L stainless steel finds wide application in marine equipment, fertilizer production equipment, and other fields [24,25]. However, some studies show that 316L stainless steel can still corrode when the corrosive ion content in the environment is too high [26,27]. Zhang et al. [28] confirmed that the preparation of Stellite6 composite coating on the surface of 316L stainless steel improved its corrosion resistance at high temperatures. For practical implementation, the overall quality of the clad layer, encompassing both forming quality and performance, is critical. Therefore, maintaining the excellent performance of the cladding layer and improving the forming effect will become an important factor limiting the practical application range of copper–cobalt composite coatings. Currently, there is relatively little research on this issue [16,29]. This research investigated the preparation of cobalt–copper alloy composite coatings using laser cladding technology by varying the copper content in composite coatings. The effects of laser power and laser scanning speed on the properties of the composite coatings were also studied to obtain better cladding layer performance and form effect. The hardness, wear resistance, and corrosion resistance of the composite coatings were significantly improved compared to the substrate, which has certain significance for expanding the practical application (such as plow teeth) of cobalt–copper alloy composite coatings. In this paper, laser powers of 800 W, 1000 W, and 1200 W and scanning speed parameters of 600 mm/min, 800 mm/min, and 1000 mm/min were used to prepare Stellite6/Cu composite coatings on the surface of 316L stainless steel, and the surface microstructure, phase composition, microhardness, wear resistance, and corrosion resistance of the coatings were tested and analyzed.

## 2. Experimental Materials and Preparation Methods

### 2.1. Experimental Materials

The plow teeth made from 316L stainless steel were used as the experimental substrate material. Stellite6/Cu composite coatings were prepared onto the surface of 316L stainless steel using laser cladding technology. High-purity copper powder and Stellite6 alloy powder were selected as cladding materials, and their chemical compositions are shown in Table 1 and Table 2, respectively.

The grain diameters of the Cu powder were 25–48 μm, and those of the Stellite6 were 45–90 μm.

### 2.2. Experimental Preparation Methods

The fiber-optic laser cladding system (Model XL- F2000T, Guangzhou xinlai laser technology Co., Ltd., Guangzhou, China) was used to deposit Stellite6/Cu powders. The laser has an output power of 0–2000 W and a wavelength of 1060–1100 nm. Laser power ranges of 800 W, 1000 W, and 1200 W, scanning speeds of 600 mm/min, 800 mm/min, and 1000 mm/min, the defocus distance of +2 mm, and the spot diameter of 4 mm were employed to prepare the samples with Stellite6/Cu composite coatings. In addition, the laser cladding method of the single-pass cladding and the multi-pass cladding was adopted. The composite coatings with different copper contents were then prepared after selecting the appropriate laser power and scanning speed based on the results.

The size of the 316L substrate was 200 mm (length) × 100 mm (width) × 50 mm (thickness). The surface of 316L was polished by sandpaper of 25 μm, 13 μm, 8.5 μm, and 6.5 μm in sequence to remove the oxide layer. After polishing, the surfaces were cleaned by 100% ethanol and then left to dry naturally. Section 3.1, Section 3.2, Section 3.3, Section 3.4 and Section 3.5 outline how laser cladding modification technology to apply Stellite6/Cu composite coatings on the surface of 316L plow teeth was studied by changing the laser power and changing the scanning speed. The weight percent of the Cu powder was 1% in the Stellite6/Cu mixing powders. And then, the effect of changes in Cu content from wt % of 1 to wt % 10 on the performance of the Stellite6/Cu composite coatings was investigated, as outlined in Section 3.6 and Section 3.7, according to the optimal process parameters. The densities of Cu, Co, and Cr are very close, with 8.96 g/cm^3^, 8.9 g/cm^3^, and 7.2 g/cm^3^, respectively, so the mixing of Stellite6/Cu is easy to achieve and does not require adhesives. The Stellite6/Cu powders were stirred mechanically by the commonly used physical methods until they were evenly mixed. Laser cladding created a 2 mm thick cladding powder layer on the surface of the 316L stainless steel.

After completing the laser cladding, a wire-cutting machine was employed to process the samples into specimens with dimensions of 50 mm × 10 mm × 5 mm and 10 mm × 10 mm × 10 mm. Cold inlay powder and cold inlay liquid were used to inlay the specimens in a ratio of 5:4. The inlayed specimens were sanded with sandpaper of 8.5–80 μm and then polished with alumina polishing agents with diameters of 5.0 μm and 1.0 μm. The polishing cross-section was studied by the following methods: a scanning electron microscope (SEM, HITACHI SU8010, Tokyo, Japan), energy dispersive spectrometer testing (EDS, Noran System 7, Thermo Fisher Scientific, Waltham, MA, USA), X-ray diffraction (XRD, SHIMADZU XRD-6100, Kyoto, Japan), a friction and wear test (SFT-2M, Lanzhou Zhongke Kaihua Technology Co. Ltd., Lanzhou, China), and electrochemical and immersion experiments testing (CHI660E, Chenhua Instrument, Shanghai, China).

## 3. Results and Analysis

### 3.1. Influence of Laser Power on the Microstructure and Phase Composition of Composite Coating

**Figure 1:**
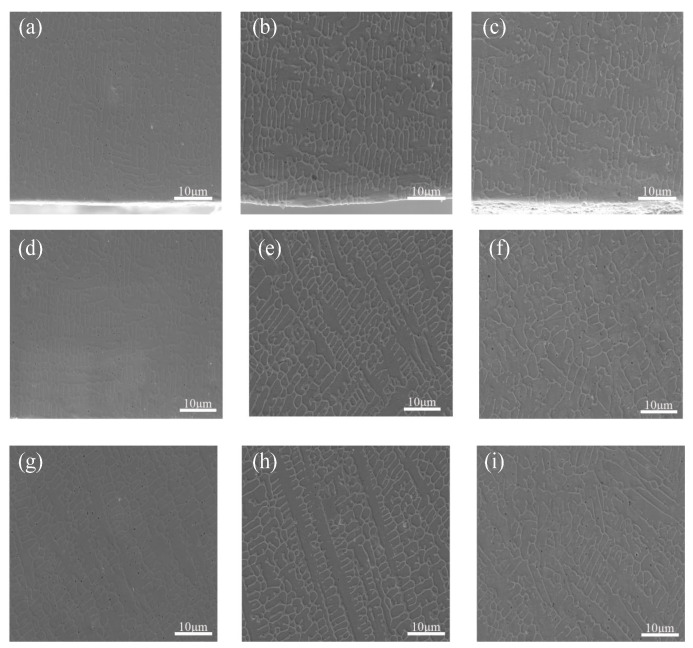
SEM images of the (**a**–**c**) top melting, (**d**–**f**) central bonding, and (**g**–**i**) bottom affecting zones of Stellite6/Cu composite coatings prepared using laser powers of (**a**,**d**,**g**) 800 W, (**b**,**e**,**h**) 1000 W, (**c**,**f**,**i**) 1200 W.

A constant scanning speed of 1000 mm/min was maintained while changing the laser power. The SEM images of the top of the Stellite6/Cu composite coatings of the specimens are displayed in Figure 1. According to the distance from the top surface of the coating, the cladding layers were divided into three zones, namely the melting zone at the top (0.2 mm to 0.4 mm from the top surface of the coating), the bonding zone in the middle (0.4 mm to 1.0 mm from the top surface of the coating), and the heat-affecting zone at the bottom (1.0 mm to 1.6 mm from the top surface of the coating).

In Figure 1, it is evident that the density of the microscopical grain structure in the Stellite6/Cu cladding layers first increased and then decreased when the laser power increased. The grain growth direction was disordered at 1000 W, indicating that the top melting zone was unevenly heated and the cooling rate fluctuated greatly, resulting in increasing stress on the grain structure dislocations, which helped to improve the hardness of the cladding layer. While the laser power was 800 W, the composite coating slowed grain structure growth and shallowed formation. The grain structure in the top melting zone was mainly composed of dendritic and columnar crystals, because the heating at the top was uneven, and the main heat dissipation method was evaporation. The significant temperature gradient difference promoted the grain structure in the top melting zone being mainly dendritic and columnar crystals. According to the theory of rapid solidification [30,31], the main factor affecting the microstructure formation of the cladding layer is the ratio of temperature gradient to growth rate. Due to the significant influence of the Marangoni effect on the top and middle zones of the cladding layers, the grain structure in these zones often appears in an amorphous growth direction. The dense grain structure formed in the top melting zone at 1000 W. Compared with the top zone, the central bonding zone showed a trend of slowing down the crystallization rate due to the cooling effect of the substrate heat dissipation. Also, the heat dissipation efficiency was fastest in the direction perpendicular to the cladding layer, and the grain structure gradually changed from a single columnar crystal to a mixed growth of columnar crystals and columnar dendrites. The heat generated by laser cladding changed from substrate heat dissipation to conduction heat dissipation at the bottom affecting zone, resulting in a decrease in heat dissipation efficiency and an extension of the grain-cooling process, presenting neatly arranged, coarse crystal branches.

**Figure 2:**
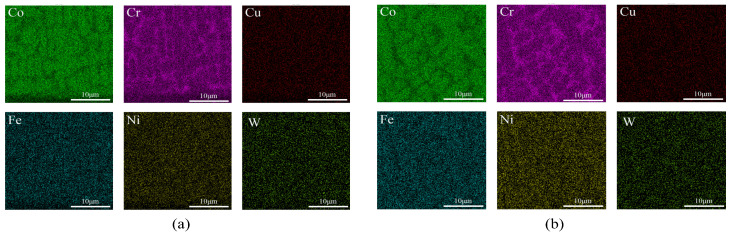
(**a**) Top and (**b**) bottom EDS surface scanning of composite coating prepared at 800 W.

**Figure 3:**
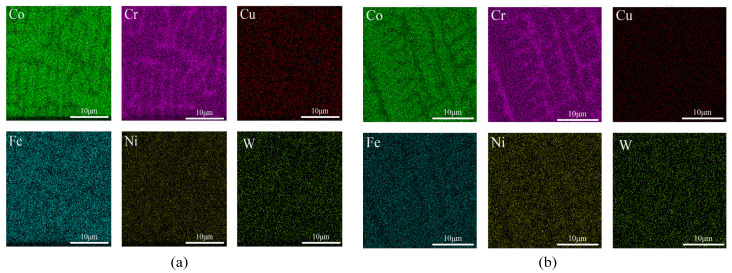
(**a**) Top and (**b**) bottom EDS surface scanning of composite coating prepared at 1000 W.

**Figure 4:**
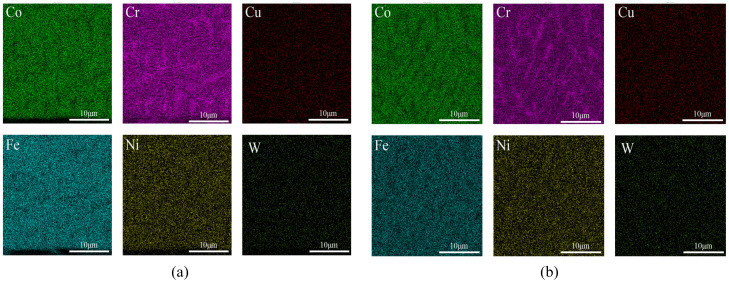
(**a**) Top and (**b**) bottom EDS surface scanning of composite coating prepared at 1200 W.

Figure 2, Figure 3 and Figure 4 show the EDS surface scan element distribution in the top melting zone and the bottom affecting zone of Stellite6/Cu composite coatings prepared at different laser powers. These images reveal that the main elements in the coating are Co, Cr, Cu, Fe, Ni, W, etc., and each element is distributed uniformly at the top of the coating. The element distribution compositions are shown in Table 3 and Table 4. Furthermore, new phases gradually appeared in the coating as the laser power increased. The Co_0.72_Fe_0.28_ phase appeared at the laser power of 1000 W, which helped to improve the hardness and wear resistance of the coatings. When the laser power was 1200 W, the Fe_0.64_Ni_0.36_ phase appeared, which had a negative effect on the hardness enhancement. Table 3 shows the top element content of the cladding layer prepared with different laser powers; as the laser power increased, the Co, Cr, Cu, and W elements at the top of the coating gradually decreased, while the Fe and Ni elements showed an increasing trend.

The element distribution content is shown in Table 4. According to Table 4, as the laser power increased, the Co and W contents at the bottom of the coating gradually decreased, while the Cr and Cu elements first increased and then decreased, and the Fe and Ni elements showed an increasing trend. From Figure 2, Figure 3 and Figure 4, it can be seen that the increased laser power led to the gradual aggregation of the Cr and Cu elements towards the heat-affected zone at the bottom of the coating, while the Fe and Ni elements aggregated to form the Fe_0.64_Ni_0.36_ phase. With the laser power increasing, the Cr and Cu elements gradually settled towards the bottom due to their large density. However, at 1200 W, the substrate was overheated and the cooling rate of the melt pool increased, resulting in the re-precipitation of Cr and Cu.

**Figure 5:**
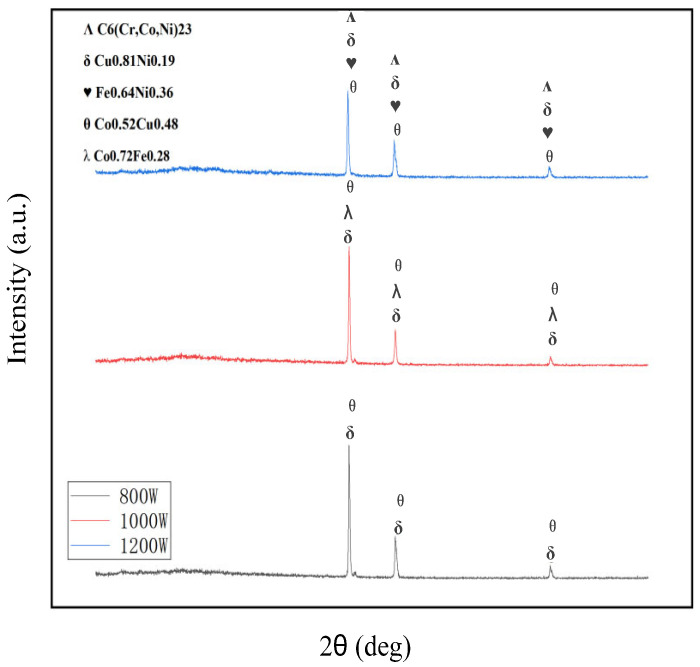
Phase components of composite coating prepared at different laser powers.

The XRD pattern of the composite coatings is shown in Figure 5. The diffraction angle range of the horizontal axis is 20–90° in Figure 5. Based on the EDS results from Figure 3 and Figure 4, and the phase composition of the coating in Figure 5, it is indicated that the increase in laser power led to the gradual aggregation of the Co, Cr, Cu, and W elements towards the heat-affected zone at the bottom of the coatings. The Fe and Ni elements aggregated to form the Fe_0.64_Ni_0.36_ phase. Due to the high density of the Co and Cu elements, they gradually deposited towards the bottom of the coating under gravity and diluted the substrate, forming the Co_0.52_Cu_0.48_ phase during this deposition process.

### 3.2. Influence of Scanning Speed on the Microstructure and Phase Composition of Composite Coating

**Figure 6:**
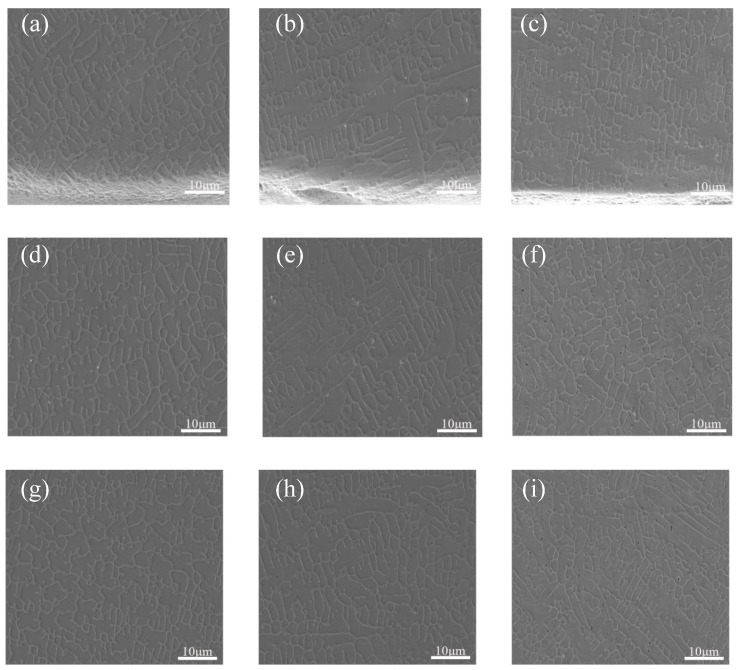
SEM images of the (**a**–**c**) top melting zone, (**d**–**f**) middle bonding zone, and (**g**–**i**) bottom affecting zone of Stellite6/Cu composite coating prepared with scanning speeds of (**a**,**d**,**g**) 600 mm/min, (**b**,**e**,**h**) 800 mm/min, and (**c**,**f**,**i**) 1000 mm/min.

A constant laser power of 1000 W was maintained while changing the scanning speed. Figure 6a–c show the SEM images of composite coating prepared at different scanning speeds. The scanning speeds, the lower the coating heights. Due to the small temperature gradient and growth rate ratio between the top and middle of the cladding layer, the coating reached a dynamic equilibrium of heating and heat dissipation, resulting in a large number of dendritic crystals appearing at the top of the cladding layer, and the direction of grain growth was not fixed.

SEM microstructures of the bonding zone at the middle and the bottom of composite coatings prepared at the scanning speeds are shown in Figure 6d–i. It is indicated in Figure 6d–f that the crystalline grain structure density in the middle bonding zone of the cladding layer increased with the theory of rapid solidification [30,31]. The crystalline grain structure in the middle zone was mainly composed of columnar crystals and columnar dendrites, exhibiting a certain growth directionality compared to the top melting zone. In Figure 6h–i, the crystalline grain structure transformed from columnar crystals and columnar dendrites to cellular crystals and elongated columnar crystals. The directional change in crystalline grain growth was pronounced compared to the middle bonding zone, suggesting that the low heat absorption efficiency of the cladding layer caused by faster scanning speeds might hinder crystalline grain formation.

**Figure 7:**
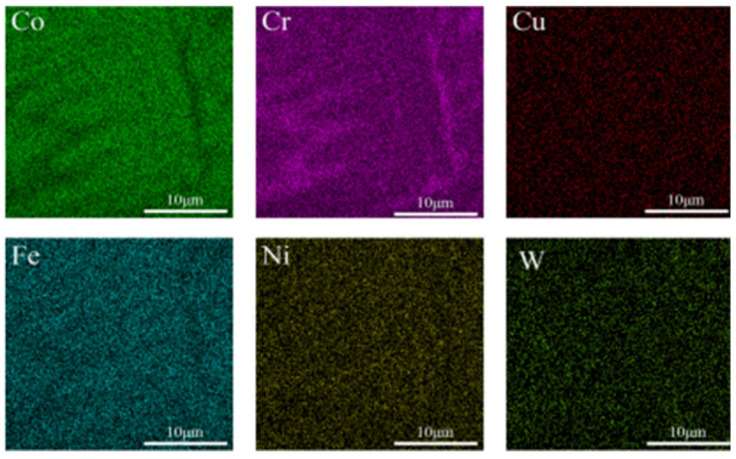
Top EDS surface scanning of composite coating prepared at 600 mm/min.

**Figure 8:**
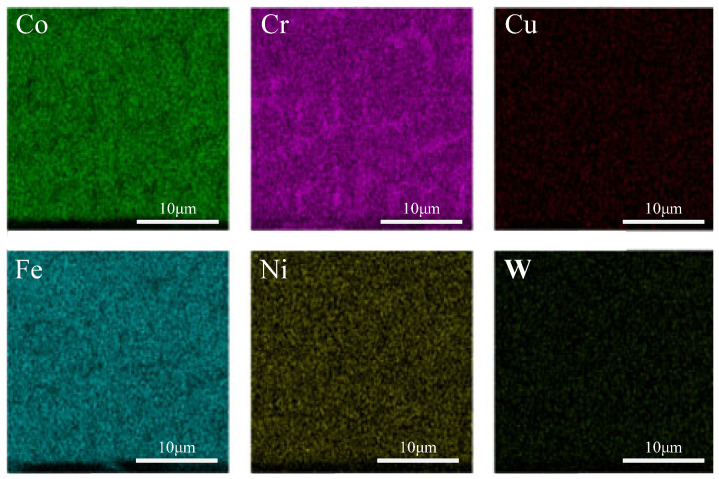
Top EDS surface scanning of composite coating prepared at 800 mm/min.

**Figure 9:**
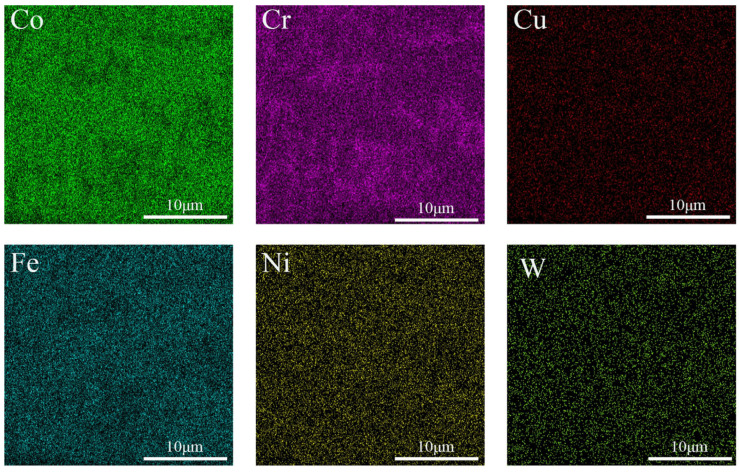
Top EDS surface scanning of composite coating prepared at 1000 mm/min.

Figure 7, Figure 8 and Figure 9 show the EDS surface scan element distribution at the top melting zones of composite coatings prepared at different scanning speeds. It can be seen that the main elements in the coating are Co, Cr, Cu, Fe, Ni, W, etc., and each element is uniformly distributed at the top of the coating. The distribution composition of these elements is shown in Table 5. The CoCx phase appeared when the scanning speed was 800 mm/min, contributing to improved corrosion resistance. According to Table 5, as the scanning speed increased, Co, Cr, and W showed a trend of first increasing and then decreasing, while the Cu, Fe, and Ni contents first decreased and then increased. This behavior is attributed to elements such as Co, Cr, and W gradually sinking under the influence of gravity, forming new phases at the bottom. Cu, Fe, Ni, and other elements gradually melted in the middle to form new phases and then re-precipitated at the bottom. Comparing Table 3, Table 4 and Table 5, it is noted that the concentration of Fe was relatively high, but the Fe content of the starring Stellite6/Cu powder was low. It indicated that the crystalline grain zone and the area between crystalline grains in the cladding composite coatings were favorable for Fe enrichment. This might be because Fe easily forms Fe-based sosoloid with Co and Cr, ultimately leading to eutectic structures.

**Figure 10:**
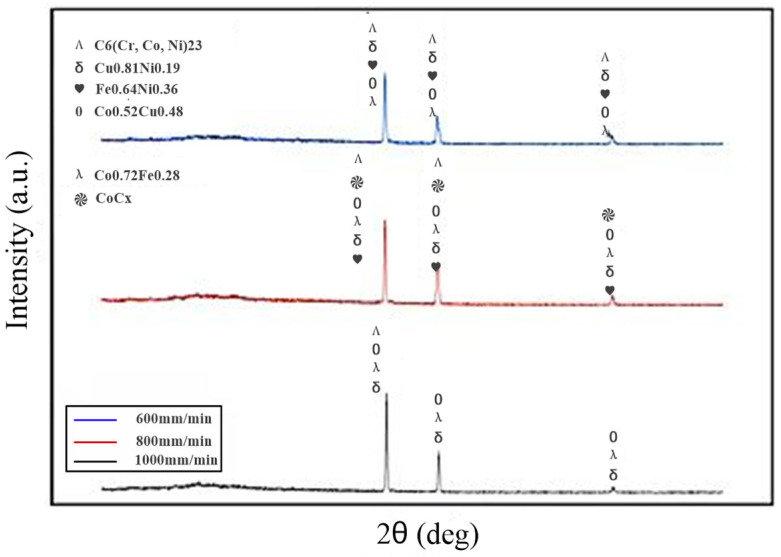
Phase components of composite coatings prepared at different scanning speeds.

Figure 10 shows the phase composition of composite coatings prepared at different scanning speeds. In Figure 10, the range of the diffraction angle was 20–90°. It can be seen that the CoCx phase only formed at a scanning speed of 800 mm/min, which improved the wear resistance and corrosion resistance of the coating.

### 3.3. Effect of Laser Power on the Microhardness and Wear Properties of Composite Coating

**Figure 11:**
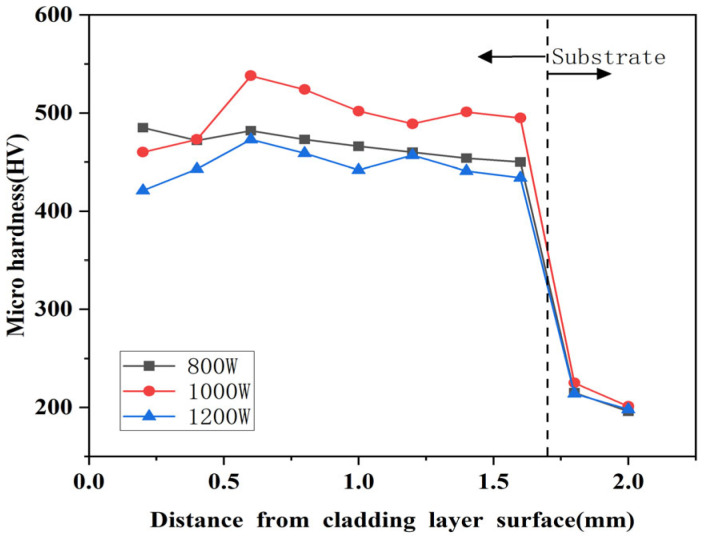
Influence of different laser powers on the hardness of the coating under 600 mm/min.

**Figure 12:**
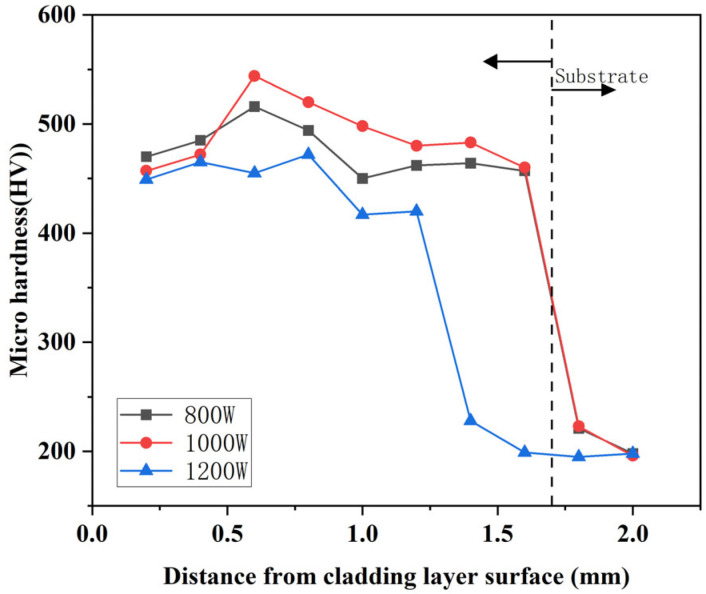
Influence of different laser powers on the hardness of the coating under 800 mm/min.

**Figure 13:**
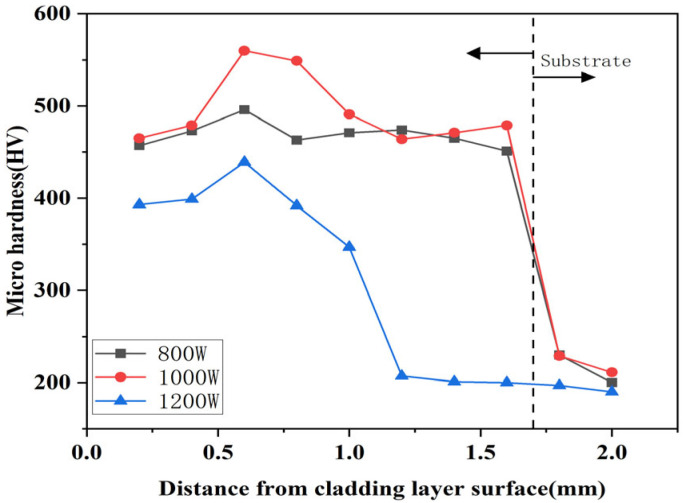
Influence of different laser powers on the hardness of the coating under 1000 mm/min.

Figure 11, Figure 12 and Figure 13 show the microhardness distribution of composite coatings prepared using different laser power parameters. According to these Figures, it can be seen that the hardness curve exhibits three stages corresponding to the top melting zone of the cladding layer (0.2–0.4 mm from the coating surface), the middle bonding zone (0.4–1.0 mm from the coating surface), and the bottom heat-affected zone (1.0–1.6 mm from the coating surface). The top melting zone mainly received direct irradiation from the laser. The middle bonding zone was greatly affected by the thermal energy generated by the laser, sufficient to elevate the austenite transformation temperature threshold, resulting in the most significant increase in hardness. The laser energy density received at the bottom of the cladding layer gradually decreased and was insufficient to cause significant change in the substrate.

From Figure 11, Figure 12 and Figure 13, it can be seen that the microhardness of the composite coating prepared using a laser power of 1000 W was superior to those prepared at 800 W and 1200 W. The average microhardness of the coating showed a trend of first increasing and then decreasing with increasing laser power, and the fluctuation amplitude of hardness was positively proportional to the increase of laser power. Comparing the Figure 11, Figure 12 and Figure 13, it can be seen that the hardness improvement was significant and fluctuations were relatively small at a scanning rate of 800 mm/min. The average hardness of the composite coating peaked at 489.25 HV when the laser power was 1000 W, approximately 1.33 times higher than the average hardness of the substrate at 209.5 HV. Furthermore, the maximum hardness appeared in the middle bonding zone of the cladding layer, which reached 544 HV, an increase of about 1.59 times compared to the substrate. As the laser power increased, the temperature of the melt pool also increased greatly, making the powder and the substrate material absorb more heat, which was beneficial for generating more hard phases inside the cladding layer. However, when the laser power reached 1200 W, excessive melt pool temperature caused hard-phase overgrowth and grain coarsening. The excessive growth of crystalline grains reduced the density of crystalline grain boundaries and the mobility of dislocations in the coating, increasing coating brittleness and susceptibility to cracking, thereby decreasing the microhardness of the coating.

**Figure 14:**
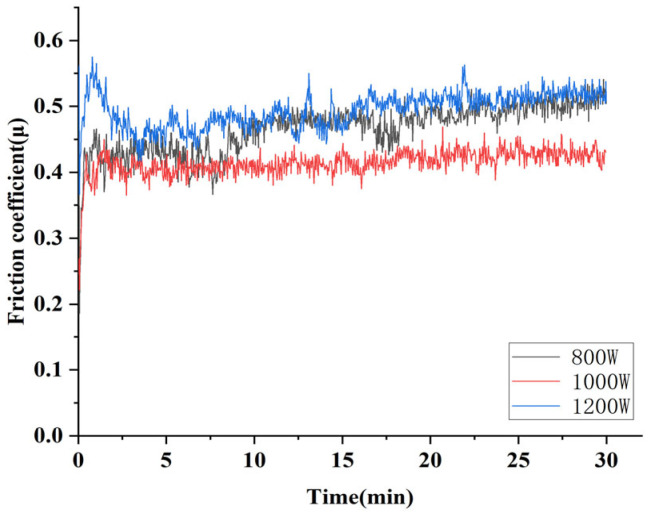
Friction coefficient of composite coatings prepared by different laser powers under Si_3_N_4_ ball.

Figure 14 shows the friction and wear coefficients of composite coating prepared by different laser powers under a 4 mm diameter Si_3_N_4_ friction ball. As shown in Figure 14, the friction and wear test requires a certain period of grinding-in before entering a stable wear period. After the grinding-in period, the surface of the sample underwent certain surface hardening, and the actual contact area between the friction ball and the sample increased, the pressure decreased, and the friction and wear coefficient began to stabilize. The coating prepared at 1000 W exhibited the lowest dilution rate and strongest grain refinement, collectively contributing to optimal wear resistance. Both factors contributed to the optimal wear resistance of the composite coating; it had a good friction coefficient, reducing by 14.3% and 17.1%, respectively, compared to coatings prepared at the laser powers of 800 W and 1200 W. The friction coefficient at 1000 W was also the most stable. Therefore, the coating prepared at 1000 W demonstrated the best wear resistance.

### 3.4. Effect of Scanning Speed on the Microhardness and Wear Properties of Composite Coating

**Figure 15:**
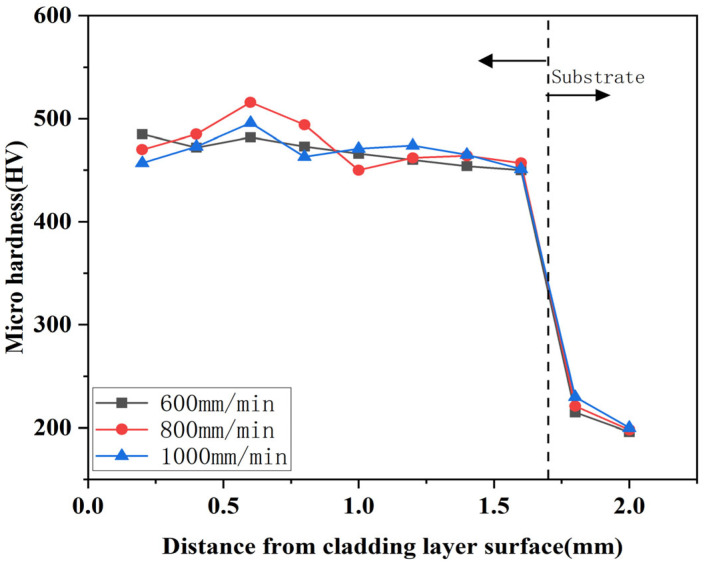
Influence of different scanning speeds on the hardness of the cladding under 800 W.

**Figure 16:**
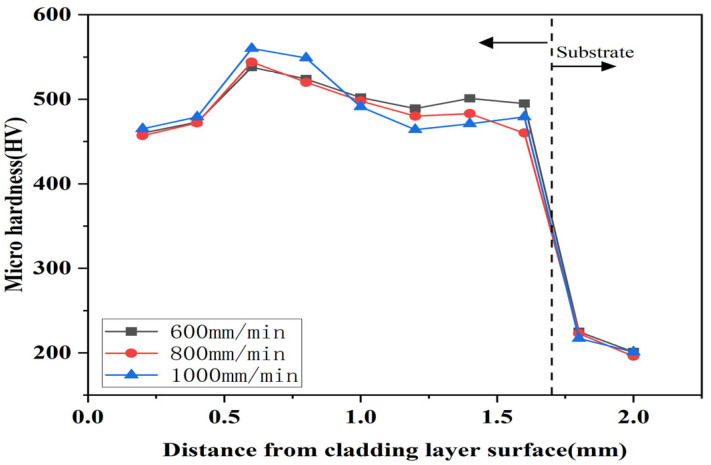
Influence of different scanning speeds on the hardness of the cladding under 1000 W.

**Figure 17:**
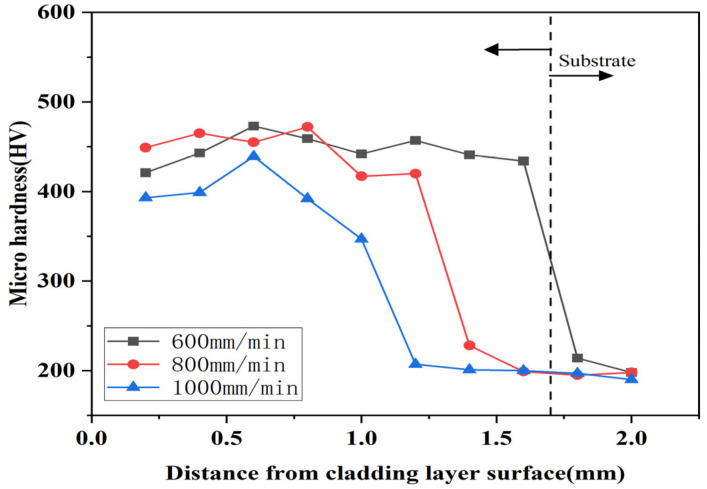
Influence of different scanning speeds on the hardness of the cladding under 1200 W.

Figure 15, Figure 16 and Figure 17 show the variation of microhardness of composite coating prepared under different scanning speeds with respect to the depth perpendicular to the longitudinal direction of the cladding layer. According to the Figures, coating microhardness increased relative to the substrate and increased then decreased with increasing scanning speed. The heat-affected zone at the junction of the substrate and the cladding layer sharply decreased, while the average hardness changed in the bottom heat-affected zone of the substrate, and the cladding layer was minimal.

Comparing the Figure 15, Figure 16 and Figure 17, it can be seen that the highest microhardness of the cladding layer occurred in the middle bonding zone at 1000 W and 1000 mm/min, and it was 560 HV, which was about 1.67 times higher than that of the substrate. Also, the microhardness was slightly higher than that under 800 mm/min, but the microhardness fluctuation was greater than that at 800 mm/min. Therefore, the parameters with 1000 W and 1000 mm/min might be more suitable for laser cladding.

Heat transfer efficiency in the melt pool depended primarily on substrate heat and laser energy density. Therefore, in order to ensure that the powder can be fully melted, and that the crystalline grain structure growth is good, the scanning speed should not be too fast or too slow when the laser power is constant. When the scanning speed was 800 mm/min, the scanning speed was suitable for slowly heating up in the melt pool. According to Figure 10 and Table 5, it can be seen that the Stellite6/Cu and the substrate melted and released elements such as Co and Cu, forming more hard-phase structures. More importantly, the temperature conditions inside the coating were optimal. Considering Figure 6d–i, it can be seen that this not only promoted rapid grain growth but also controlled its duration, preventing excessive grain expansion. In this environment, Stellite6 played a role in refining the crystalline grain structure, further promoting the refinement of the microstructure. When the surface of the coating was subjected to a certain load, the internal hard phase could effectively reduce the deformation and crack propagation trend of the coating due to increased microhardness.

**Figure 18:**
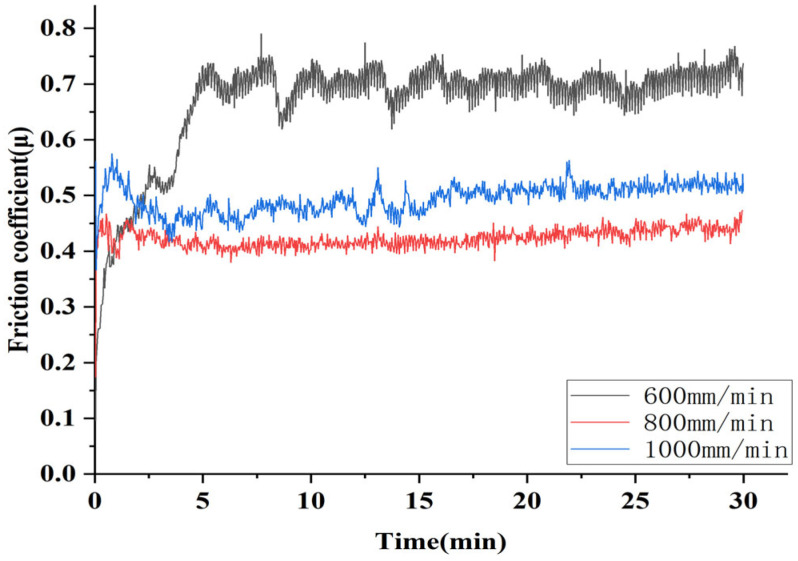
Friction coefficient of composite coating prepared by different scanning speeds under Si_3_N_4_ ball.

Figure 18 shows the wear property using Si3N4 friction balls. According to Figure 18, the average friction coefficients were 0.706 (600 mm/min), 0.437 (800 mm/min), and 0.515 (1000 mm/min); the coating prepared at 600 mm/min exhibited strong fluctuation during wear, whereas those at 800 mm/min and 1000 mm/min fluctuated less. Therefore, the results indicated that the wear resistance of the Stellite6/Cu composite coating reached its optimum at a scanning speed of 800 mm/min. The average friction coefficient of the coating at a scanning speed of 800 mm/min decreased by 35.39% and 15.1% compared to 600 mm/min and 1000 mm/min, respectively. From this, it could be concluded that the Stellite6/Cu composite coating prepared at a scanning speed of 800 mm/min exhibited the best wear resistance. Based on Figure 6d–i and Figure 18, it could be concluded that the dilution rate of the cladding layer prepared at a scanning speed of 800 mm/min exhibited the best wear resistance, and the degree of crystalline grain refinement was the strongest, which together created the optimal wear resistance of the coating.

### 3.5. Effect of Scanning Speed on the Corrosion Resistance of Composite Coating

**Figure 19:**
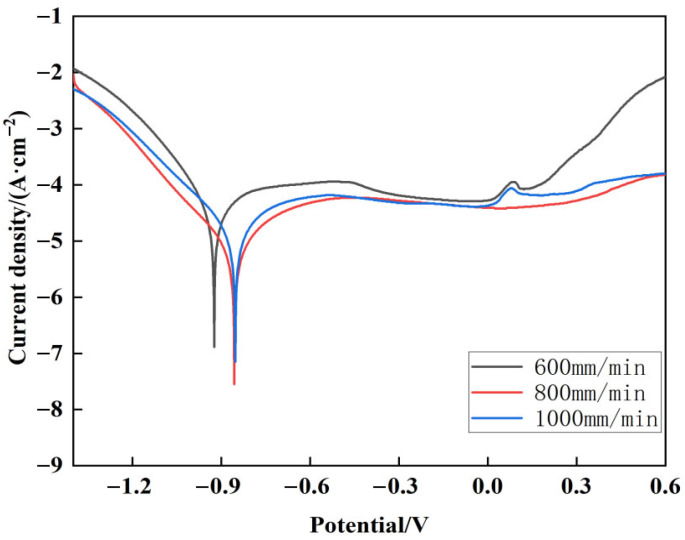
Potentiodynamic polarization curves of composite coatings with different scanning speeds.

Figure 19 shows the potential polarization curves of Stellite6/Cu composite coatings prepared at different scanning speeds after electrochemical corrosion in an NaCl solution with a 3.5 wt %. Cathodic and anodic reactions occurred as described [32]. The electrochemical results of composite coatings prepared at different scanning speeds are listed in Table 6. The self-corrosion current densities of the composite coatings decreased then increased with increasing scanning speed.

According to Table 6, the self-corrosion current densities of composite coatings prepared at different scanning speeds were −6.89 µA/cm^2^, −7.55 µA/cm^2^, and −7.15 µA/cm^2^, and the self-corrosion potentials were −924 mV, −856 mV, and −853 mV. The composite coating prepared at 800 mm/min exhibited the lowest current density, while its self-corrosion potential was similar to that at 1000 mm/min. In addition, the passivation region at 800 mm/min was relatively wide and flat, with no pitting peaks observed between −0.3 V and 0.3 V, indicating superior corrosion resistance. The corrosion trend of metal in halogen environments is inversely proportional to their self-corrosion potential, and the corrosion rate is directly proportional to the current density. Taking into account the corrosion tendency and corrosion rate of metal, the corrosion resistance of alloy cladding layers can be reflected [33,34]. Therefore, considering both tendency and rate, the coating prepared at 800 mm/min demonstrated the best corrosion resistance due to its low corrosion rate and low corrosion tendency.

### 3.6. Effect of Cu Content on the Microhardness and the Friction Behavior of Composite Coating

**Figure 20:**
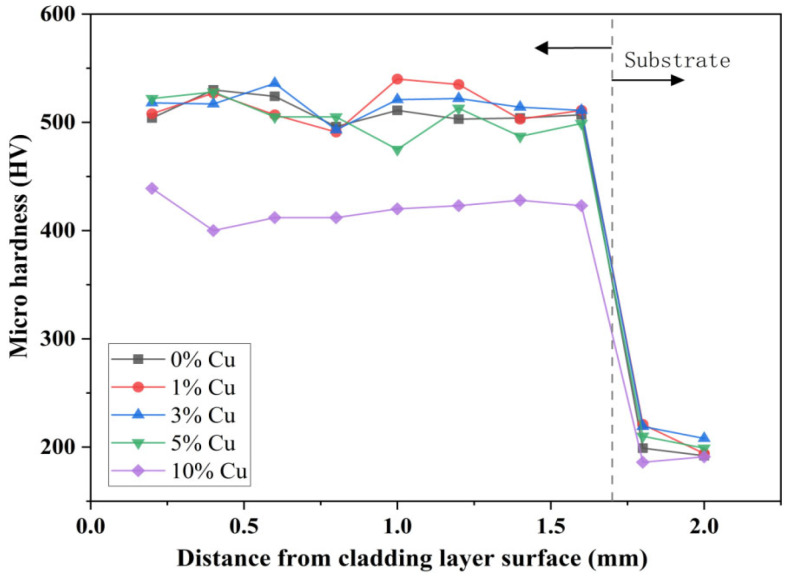
Hardness distribution of copper-based alloy cladding layer with different Cu weight percents.

Based on previous results, Stellite6/Cu composite coatings with varying copper content (1, 3, 5, 10 wt.%) were prepared using 1000 W and 800 mm/min, and then the composite coatings were tested. The hardness test results are shown in Figure 20. The detection points were perpendicular to the longitudinal direction of the cladding layer, with a spacing of 0.2 mm between adjacent detection points. The 316L substrate was located at a distance of 1.6 mm or more from the surface of the coating. From Figure 20, it can be seen that the microhardness change trend of cladding layers prepared with different copper content was generally similar: the maximum hardness appeared in the middle of the cladding layer, and the Vickers hardness reached 2.1~2.6 times that of the substrate. After entering the heat-affected zone, the hardness began to sharply decrease, indicating that the hardness of the heat-affected zone was not affected by the weight percent of the copper-based alloy. It can be found that the microhardness of the substrate zone decreased again compared to the heat-affected zone, and the hardness stabilized at 195 HV. Compared to Ref. [12], adding a certain copper into Stellite6 enhanced the hardness more effectively than adding WC. The best hardness when adding WC was less than twice that of the stainless steel substrate, and similar results were obtained in Ref. [22]. Moreover, as mentioned in Ref [16], optimal Cu content refines microstructures and promotes nanocrystal formation.

**Figure 21:**
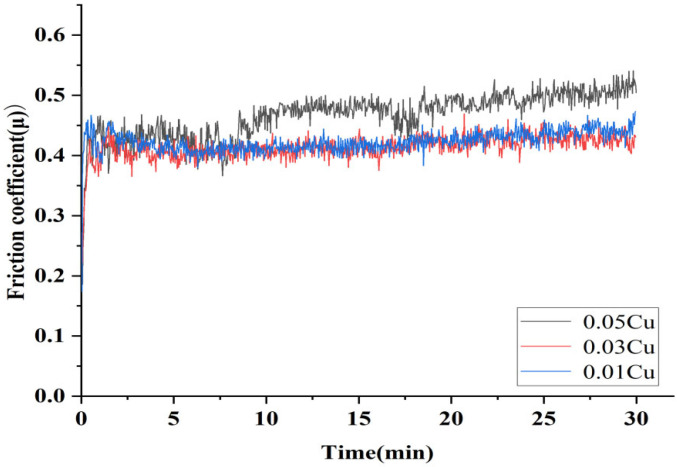
Friction coefficient of composite coatings prepared at the different weight percents of copper.

Figure 21 shows the wear properties of composite coatings prepared by adding Cu with different weight percents. From the Figure, it can be seen that the friction ball and the surface of the tested sample gradually became stable as the friction and wear test progressed. The SEM results of 10 wt.% copper content were very poor, so this weight percent was directly removed from the friction experiment. The average friction coefficient of the composite coating during the stable period (20–30 min) was calculated. When the copper contents were 1%, 3%, and 5%, the average friction coefficient were 0.424, 0.452, and 0.431, respectively. Therefore, the coating with 1 wt.% Cu exhibited the best wear resistance.

### 3.7. Effect of Cu Content on the Corrosion Resistance of Stellite6/Cu Composite Coating

**Figure 22:**
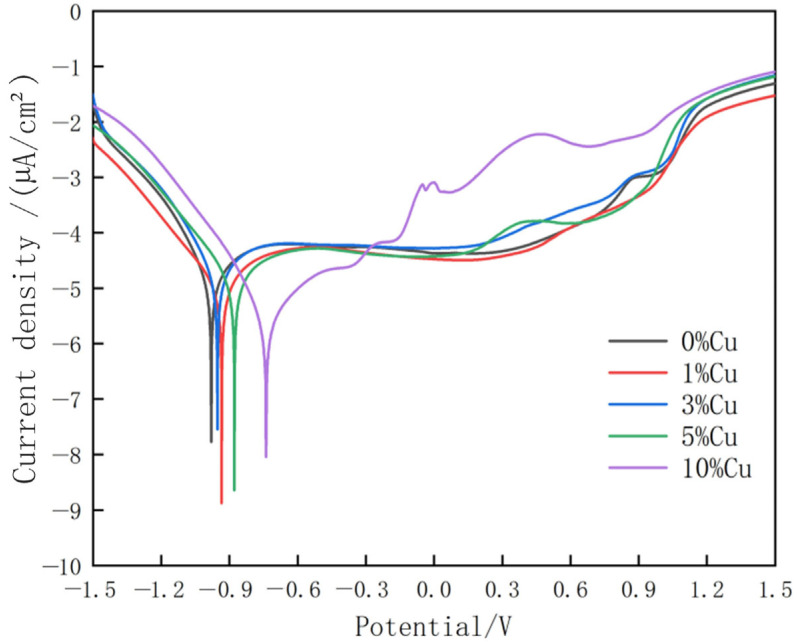
Potentiodynamic polarization curves of composite coatings with different weight percents of copper.

Corrosion resistance testing of composite coatings containing copper is necessary due to the inherent corrosion resistance of copper [3]. Therefore, Figure 22 shows the self-corrosion potential polarization curves of Stellite6/Cu composite coating prepared at different Cu weight percents after electrochemical corrosion in an NaCl solution with a 3.5 wt.%, and the specific values are shown in Table 7. In NaCl solution, the self-corrosion current density reached its minimum value (−8.878 µA/cm^2^) at 1 wt.% Cu. The self-corrosion potential was the criterion for judging the corrosion resistance tendency, while the self-corrosion current density was the key criterion for judgment. The corrosion rate of metals in halogen environments is directly proportional to the self-corrosion current density. The results indicated that the copper-based alloy cladding layer with a 1 wt.% exhibited the slowest corrosion rate and best corrosion resistance.

## 4. Conclusions

Stellite6/Cu alloy composite coatings on 316L stainless steel surfaces prepared by the laser cladding method to enhance the performance of plow teeth were studied. The main conclusions are as follows:

(1) Under excessively low laser power or high scanning speed conditions, low laser energy density resulted in poor coating formation quality.
(2) With increasing laser power, the Co element gradually accumulated at the top of the coating, while the Cr and Cu elements gradually gathered towards the heat-affected zone at the bottom of the coating. However, at 1200 W, substrate overheating and increased melt pool cooling rates caused Cr and Cu to re-precipitate.
(3) With increasing scanning speed, the Cu element gradually accumulated towards the heat-affected zone at the bottom of the coating, while the Co and Cr elements gradually transferred from the middle of the coating to the top, but the Co and Cr elements re-precipitated at the bottom of the coating at 1000 mm/min.
(4) The coating prepared at 1000 W and 800 mm/min exhibited optimal microhardness, wear resistance, and corrosion resistance.
(5) The composite coating with 1 wt.% Cu achieved the highest hardness, best wear resistance, and best corrosion resistance, effectively improving the surface performance of 316L plow teeth.

This research demonstrates the practical value of improving the surface performance of 316L stainless steel.

## Figures and Tables

**Table 1:** Chemical composition of high-purity copper powder.

Materials	Sn	Cr	S	Fe	Pb	As	Sb	Bi	Cu
wt %	<0.001	<0.001	0.002	<0.001	<0.001	<0.001	<0.001	<0.001	Balance

**Table 2:** Chemical composition of Stellite6 alloy powder.

Materials	C	Ni	W	Si	Mo	Fe	Cr	Co
wt %	1.40	3.00	8.25	1.45	1.00	3.00	29.50	Balance

**Table 3:** Element content at the top of the cladding layer prepared at different powers.

Laser Power (W)	Co	Cr	Cu	Fe	Ni	W
800	51.5	28.1	4.1	4.9	2.6	4.2
1000	46.1	25.3	3.7	8.3	3.1	4.0
1200	38.4	24.1	3.5	16.9	4.1	3.4

**Table 4:** Element content at the bottom of the cladding layer prepared at different powers.

Laser Power (W)	Co	Cr	Cu	Fe	Ni	W
800	49.6	26.1	3.8	3.9	2.6	4.2
1000	48.7	27.4	2.4	7.5	3.1	3.8
1200	39.4	25.2	3.8	18.3	4.3	3.3

**Table 5:** Element content at the top of the cladding layers prepared at the different scanning speed.

Scanning Speed (mm/min)	Co	Cr	Cu	Fe	Ni	W
600	29.4	23.0	2.5	27.6	5.3	2.5
800	44.6	25.4	2.3	11.0	3.5	3.8
1000	38.4	24.1	3.5	16.9	4.1	3.4

**Table 6:** Self-corrosion potential and current density of composite coatings prepared at different scanning speeds.

Scanning Speed	600 mm/min	800 mm/min	1000 mm/min
self-corrosion potential (mV)	−924	−856	−853
current density (µA/cm^2^)	−6.89	−7.55	−7.15

**Table 7:** Self-corrosion potential and current density of Stellite6/Cu composite coatings with different wt% of copper.

wt % of Cu	0	1	3	5	10
self-corrosion potential (mV)	−980	−934	−953	−878	−739
current density (µA/cm^2^)	−7.755	−8.878	−7.547	−8.648	−8.045

## Data Availability

The original contributions presented in this study are included in the article. Further inquiries can be directed to the corresponding author.
